# Single-molecule spectroscopy exposes hidden states in an enzymatic electron relay

**DOI:** 10.1038/ncomms9624

**Published:** 2015-10-15

**Authors:** Iris Grossman, Haim Yuval Aviram, Gad Armony, Amnon Horovitz, Hagen Hofmann, Gilad Haran, Deborah Fass

**Affiliations:** 1 Department of Structural Biology, Weizmann Institute of Science, Rehovot 7610001, Israel; 2 Department of Chemical Physics, Weizmann Institute of Science, Rehovot 7610001, Israel

## Abstract

The ability to query enzyme molecules individually is transforming our view of catalytic mechanisms. Quiescin sulfhydryl oxidase (QSOX) is a multidomain catalyst of disulfide-bond formation that relays electrons from substrate cysteines through two redox-active sites to molecular oxygen. The chemical steps in electron transfer have been delineated, but the conformational changes accompanying these steps are poorly characterized. Here we use single-molecule Förster resonance energy transfer (smFRET) to probe QSOX conformation in resting and cycling enzyme populations. We report the discovery of unanticipated roles for conformational changes in QSOX beyond mediating electron transfer between redox-active sites. In particular, a state of the enzyme not previously postulated or experimentally detected is shown to gate, via a conformational transition, the entrance into a sub-cycle within an expanded QSOX kinetic scheme. By tightly constraining mechanistic models, smFRET data can reveal the coupling between conformational and chemical transitions in complex enzymatic cycles.

## Abstract (web summary)

A major challenge in following electron transfer through dithiol/disulfide exchange is the dearth of accompanying spectroscopic effects. Here, the authors use single-molecule Förster resonance energy transfer experiments to illuminate disulfide bond rearrangements within the enzyme quiescin sulfhydryl oxidase.

## Graphical abstract

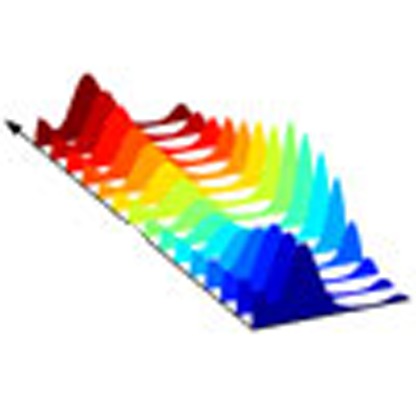

The enzyme quiescin sulfhydryl oxidase (QSOX) uses a thiol-based electron relay to generate and transfer disulfide bonds to substrate proteins^1^. QSOX, a fusion of a protein disulfide isomerase (PDI)-like oxidoreductase^2^ module and an Erv family sulfhydryl oxidase module^3^, contains two distinct redox-active sites. The intramolecular relay of electrons between these sites resembles the multiprotein relays that drive oxidative protein folding in the endoplasmic reticulum (ER) and mitochondria of eukaryotic cells and in the bacterial periplasm^4^. Although similar in biochemical function to certain ER-localized enzyme cascades, QSOX is the only secretory pathway disulfide catalyst found downstream of the ER. Mammalian QSOX is Golgi-localized in most cells, but the enzyme is upregulated and secreted from quiescent fibroblasts^5^, where it participates in extracellular matrix assembly^6^. QSOX is also found in blood and glandular secretions^7^,^8^, but little is known about its biological role in these environments. These atypical physiological contexts for a disulfide catalyst may require unique mechanistic features in QSOX. Crystallographic and biochemical analyses of mammalian and trypanosome QSOX enzymes suggested large-scale conformational changes during the reaction cycle, enabled by a flexible linker between the oxidoreductase and sulfhydryl oxidase modules^9^. In some steps of the QSOX cycle, the two modules may perform their functions independently, but the modules undergo a transient covalent linkage during electron transfer from one domain to the other^10^.

Participating in the QSOX electron relay are two redox-active di-cysteine (CXXC) motifs and a flavin adenine dinucleotide (FAD) cofactor^1^,^9^,^10^,^11^ (Fig. 1). Electrons derived from oxidation of cysteine pairs in substrate proteins first reduce the QSOX CXXC motif in a thioredoxin fold (Trx) domain of the PDI module. Electrons are then shuttled by dithiol/disulfide exchange to the second CXXC, in the Erv module, and from there to the adjacent FAD cofactor. The FAD is reoxidized by O_2_, generating H_2_O_2_ as the byproduct of disulfide bond formation. Previous studies of the QSOX mechanism followed redox events involving the FAD by changes in absorbance^10^,^12^. However, changes in protein conformation or disulfide connectivity not involving the FAD were invisible.

Structural data showing the role of protein conformation in assembling the components of the QSOX electron relay^9^,^11^ led us to inquire how conformational dynamics govern electron transfer. In particular, we sought to determine the contribution of the interdomain electron-transfer intermediate to the kinetics of turnover. A major challenge in following electron transfer through dithiol/disulfide exchange reactions is the dearth of accompanying spectroscopic effects. A practical consequence of dithiol/disulfide exchange, however, is a change in covalent connectivity within the polypeptide. This change inspired others to use single-molecule force-distance measurements to follow cysteine rearrangements in model substrate proteins^13^,^14^. We considered whether changes in covalent connectivity might affect conformational dynamics in QSOX and provide a means of tracking interdomain electron transfer. Here we use single-molecule FRET (smFRET) to measure the conformational distribution in resting and cycling QSOX. We apply these smFRET measurements, in combination with bulk QSOX catalytic assays, as constraints for an expanded mechanistic model that quantitatively links conformational transitions with chemical steps in the QSOX mechanism. This novel application of smFRET is an important contribution to efforts made over many years to determine how conformational changes promote electron transfer in disulfide shuttles^15^. Our findings regarding the effects of conformational dynamics on flux through a multi-step reaction scheme are applicable, however, to other enzymes beyond those engaged in thiol-based electron relays. QSOX thus provides a paradigm for how smFRET experiments can enrich enzyme mechanistic models.

## Results

### Production of an enzyme reporter system

Recombinant *Trypanosoma brucei* QSOX (TbQSOX), which contains seven disulfides and no unpaired cysteines, was labelled site-specifically with donor and acceptor fluorophores at additional, non-native cysteines introduced by mutagenesis (Fig. 2a). Mammalian QSOX enzymes have similar substrate specificities and steady-state enzyme parameters as TbQSOX^10^,^12^ but are burdened with native free cysteines that complicate labelling (unpublished observations). The donor fluorophore Alexa 488 C5 maleimide was positioned at residue 77, 116, 142, 147, 160 or 189 in the TbQSOX Trx domain, paired with the acceptor fluorophore Alexa 594 C5 maleimide at residue 243 in the Erv domain (Fig. 2b, Supplementary Fig. 1). These enzyme variants were designated TbQSOX D-77, D-116, D-142, D-147, D-160, and D-189, according to the donor label position. All labelled enzymes were active on the model substrate dithiothreitol (DTT; Supplementary Fig. 2).

### TbQSOX displays two distinct conformational states

Single-molecule FRET experiments were conducted on freely diffusing TbQSOX molecules. For each labelled enzyme, with one exception, FRET efficiency histograms showed two distinct populations. A dominant peak was observed at relatively low FRET efficiency values (∼0.3), and a minor peak was generally seen at higher FRET efficiencies, ranging from 0.40 to 0.93 (Fig. 2c). The dwell time in each state was on the order of the burst duration (Supplementary Fig. 3).

Although the presence of two persistent protein populations was unexpected, TbQSOX crystal structures^9^ provided a framework for assigning the two species. Fluorophore distances calculated from the FRET efficiencies of the minor species were in agreement with distances estimated^16^ from a TbQSOX structure containing a disulfide between the redox-active sites of the Trx and Erv domains, designed to represent the electron-transfer intermediate (Supplementary Table 1). We discovered, however, that this conformation, hereafter referred to as ‘closed,' does not actually require an interdomain disulfide; a D-142 mutant lacking the Trx CXXC cysteines (AXXA) and thus unable to form the interdomain disulfide nevertheless exhibited the minor, high FRET efficiency population (Fig. 2d). The major, low FRET efficiency form, the ‘open' ensemble, likely represents the rapid sampling of different relative orientations of the two TbQSOX modules connected by the flexible linker (Fig. 2e). Indeed, fluorescence-correlation spectroscopy revealed fluctuations in FRET efficiency on a submicrosecond timescale in D-116 (Supplementary Fig. 4), indicating that TbQSOX undergoes fast internal motions in addition to the slower transitions between open and closed conformers. For all paired label positions, the apparent average distances between donor and acceptor in the dynamic, open state were greater than the dye pair Förster distance of ∼54 Å (see Methods and Supplementary Table 1). In D-189, the donor fluorophore is near the linker (Fig. 2a), such that the donor–acceptor distance in this variant is relatively independent of the orientations of the two modules, yielding indistinguishable peak FRET efficiency values for the open and closed populations.

### Substrate addition repartitions TbQSOX populations

**Figure 1:**
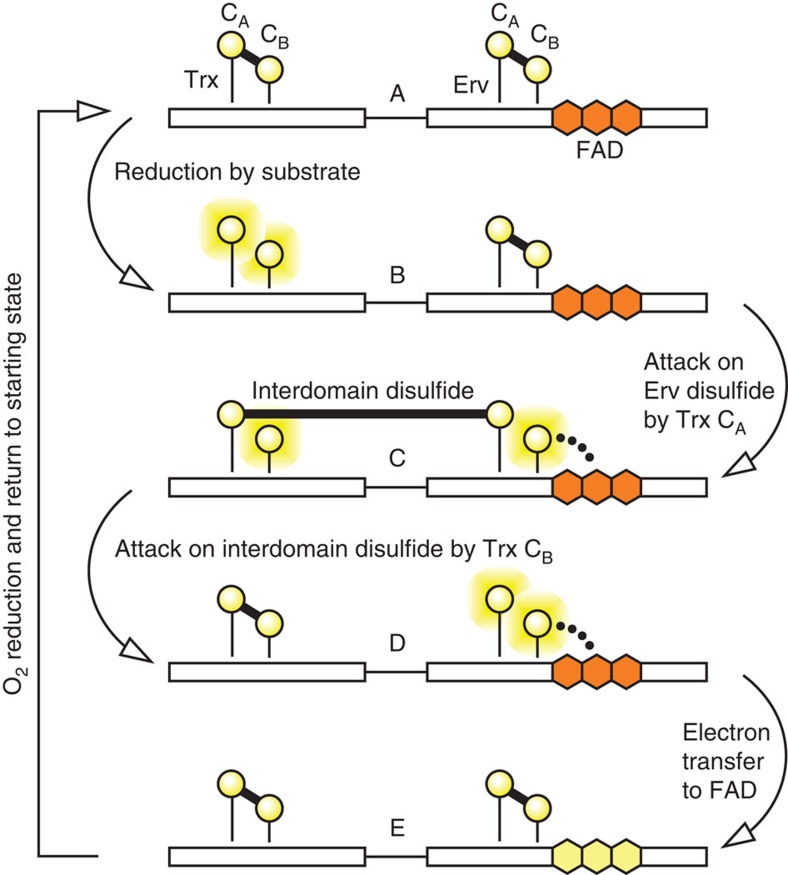
Reaction scheme of the enzyme QSOX from previous spectroscopic studies. Each functional module of QSOX contains a pair of redox-active cysteines (yellow circles). Reduced cysteines are shown with yellow halos. The FAD cofactor (orange hexagons when oxidized, yellow hexagons when reduced) is bound within the Erv domain. Heavy lines represent disulfide bonds. Dots represent a charge-transfer species. Arrows indicate transitions between states. Adapted with permission from ref. 12. Copyright (2010) American Chemical Society.

**Figure 2:**
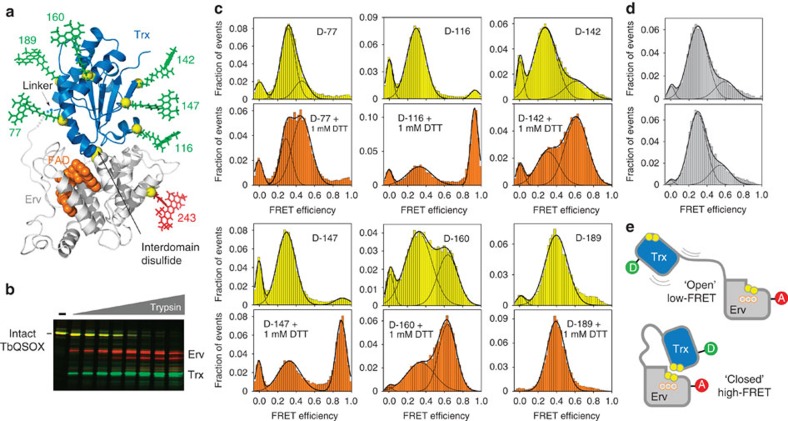
TbQSOX exhibits two conformations according to single-molecule FRET experiments. (**a**) Crystal structure of TbQSOX in the ‘closed' conformation (PDB ID: 3QD9) with the Trx domain marine blue and the Erv module grey. The FAD cofactor is orange space-filling format. Yellow spheres mark Cα atoms of residues mutated to cysteine for labelling. Fluorescent dyes are shown at their approximate positions for illustration. The figure superposes six separate double-mutants, each possessing an acceptor fluorophore at position 243 and a donor fluorophore at one of the positions labelled in green. (**b**) SDS–PAGE of a limited proteolysis experiment demonstrating that the donor fluorophore is on the Trx domain and the acceptor is on the Erv domain in TbQSOX D-116 (see Methods). For other variants see Supplementary Fig. 1a. (**c**) FRET efficiency histograms without substrate (yellow) and in the presence of 1 mM DTT (orange). Fits to up to three Gaussians are shown for illustration. Peaks at zero FRET efficiency represent molecules without an active acceptor. D-160 reproducibly showed a larger high FRET peak in the absence of substrate compared to the other variants but displayed a similar distribution between conformers in the presence of substrate as seen for the other variants. (**d**) FRET efficiency histograms of D-142 with a CXXC-to-AXXA mutation in the Trx domain active site, measured in the absence of substrate and in the presence of 1 mM DTT. (**e**) Schematic depiction of TbQSOX open and closed conformations represented by low and high FRET efficiency populations, respectively.

When the substrate DTT was added, at a concentration ∼20-fold above its apparent Michaelis constant (*K*_M_)^12^, the population distributions of the labelled TbQSOX variants at steady state (Supplementary Fig. 5) shifted markedly toward the closed conformer (Fig. 2c), such that approximately two-thirds of each protein variant population exhibited the higher FRET efficiency. The addition of DTT at this concentration had no effect on the photophysical properties of the fluorescent dyes (Supplementary Fig. 6). Although authentic TbQSOX substrates are unknown, the observed shift to a closed conformation is relevant to any substrate, as electron transfer between domains occurs downstream of electron transfer from substrate to enzyme (Fig. 1). A correlation between enzyme activity and closed conformer fraction was evident when varying the solution pH (Supplementary Fig. 7). Notably, the D-142 AXXA mutant failed to shift toward the closed conformer upon addition of substrate (Fig. 2d), despite retaining the minor, closed population as in the absence of substrate, demonstrating that a chemically competent Trx domain is required for the population shift.

**Figure 3:**
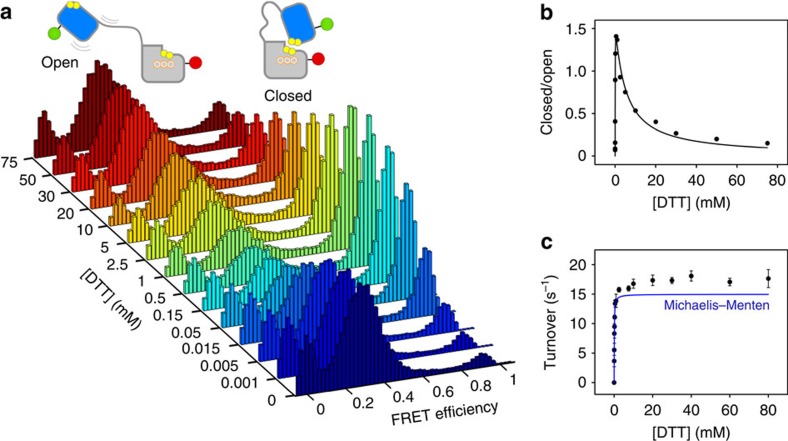
Biphasic behavior in TbQSOX conformational ensemble and catalysis. (**a**) TbQSOX D-116 FRET efficiency histograms at increasing substrate concentrations. (**b**) Ratios of TbQSOX closed and open species. For illustrative purposes, the curve is a fit to an equation of the form *A*[DTT]/(1±*B*[DTT]±*C*[DTT]^2^), corresponding to Haldane's equation for substrate inhibition^17^. (**c**) Bulk TbQSOX turnover. Error bars represent s.d. of the mean of three measurements. The blue curve represents an extension to 80 mM DTT of the Michaelis–Menten model obtained by fitting the TbQSOX turnover data from 0 to 1 mM DTT.

To quantify the effect of substrate on TbQSOX conformation, FRET efficiency histograms were next collected over a range of substrate concentrations. This and most subsequent experiments were performed using D-116, the variant showing the greatest separation in FRET efficiencies for the closed and open conformations. No additional conformations beyond those already evident in the resting enzyme were detected, but the ratio of closed to open populations increased as a function of substrate concentration up to ∼0.5–1.0 mM DTT (Fig. 3a,b). A fit to a saturation isotherm up to 1 mM DTT (Supplementary Fig. 8) yielded an apparent *K*_d_ of 40±5 μM for the transition to higher FRET efficiency, comparable to the *K*_M_ values of 65±10 μM for O_2_ consumption obtained from bulk enzyme assays in our hands (Supplementary Fig. 8) and 43 μM reported previously^12^. Surprisingly, however, at higher substrate concentrations, the amount of closed species began to decrease, indicating a second trend in the dependence of this population on substrate concentration, resembling the phenomenon of substrate inhibition^17^ (Fig. 3a,b). The midpoint of this second phase occurred at about 5 mM DTT, and by 75 mM DTT the closed conformation had dropped back nearly to the level observed in the absence of DTT.

Although depletion of the closed species with increasing substrate was so conspicuous in smFRET histograms, it did not correspond to any previously proposed transition in the TbQSOX mechanism. In contrast to the fraction of closed molecules, the turnover number of TbQSOX in bulk enzyme assays showed no decrease up to at least 80 mM DTT (Fig. 3c), ruling out substrate inhibition or damage to the enzyme as DTT concentrations surpassed 1 mM. Instead, turnover increased significantly beyond the expected values calculated from the Michaelis–Menten fit to the data up to 1 mM DTT (Fig. 3c and Supplementary Fig. 8). The second phase of increasing activity occurred in the same DTT concentration range (approximately 2–20 mM) that characterized the plunge of the closed population in smFRET, hinting that the two phenomena may be linked to the same feature of the TbQSOX mechanism. We therefore investigated whether novel insights into the TbQSOX cycle could be attained by addressing this phenomenon in an updated mechanistic model for the enzyme.

### An enhanced model for TbQSOX turnover

**Figure 4:**
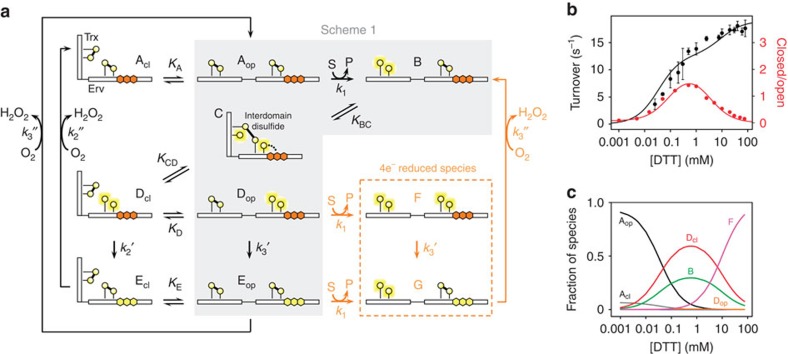
Expanded scheme for TbQSOX and steady-state concentrations of enzyme species. (**a**) States depicted in Fig. 1 are indicated on a shaded background labelled ‘scheme 1.' State C and additional proposed closed states (A_cl_, D_cl_ and E_cl_) are represented as bent forms with the two redox-active di-cysteine motifs juxtaposed. Open states (A_op_, B, D_op_, E_op_, F and G) are represented as extended forms. ‘S' and ‘P' refer to substrate (for example, DTT) and product (for example, oxidized DTT; *trans*-4,5-dihydroxy-1,2-dithiane), respectively. Rate constants for substrate oxidation, FAD reduction, and O_2_ reduction are designated. Equilibrium constants are designated as well. Rate and equilibrium constant values are reported in Supplementary Table 2. FAD reduction and O_2_ reduction are designated in the figure as two separate steps (for example, *k*_2_′ and *k*_2_″, respectively), but were fitted and reported in Supplementary Table 2 as a single value (*k*_2_), for simplicity. Other symbols correspond to Fig. 1. Additional feasible transitions not depicted in the image are reduction of A_op_ to D_op_ and reduction of B to F, but these events are negligible in wild-type TbQSOX. (**b**) Global fit of equations (4) and (5) (see Methods) to TbQSOX smFRET (red) and bulk turnover (black) data. Error bars represent s.d. of the mean of three measurements. (**c**) Steady state distribution of TbQSOX chemical/conformational states as derived from the global fit.

To account for the observations reported above, an expanded scheme for the TbQSOX mechanism was developed (Fig. 4a and Methods). Importantly, a number of other tested schemes were ruled out by the data (Supplementary Fig. 9–11). In developing the TbQSOX model, we realized that, analogous to the conformational transition A_op_↔A_cl_ observed in TbQSOX in the absence of substrate, a conformational exchange, D_op_↔D_cl_, may occur after the interdomain disulfide (state C) converts to the two-electron reduced state D (Fig. 1). As a consequence, a branch-point was introduced at state D (Fig. 4a). One potential process is continuation to E_cl_ and reaction with O_2_ to complete a catalytic cycle. Other possible fates are transition to open enzyme states and ‘capture' by excess electrons. Specifically, states F and G were introduced to represent a second round of reduction of the Trx CXXC before TbQSOX ejects its first set of electrons from the Erv domain. F and G were assigned as open conformations because they arise by reduction of open species and are likely to remain open due to electrostatic repulsion between the two reduced, negatively charged active sites in each molecule.

The expanded TbQSOX model was evaluated against the smFRET and bulk turnover data using a steady-state approximation. Fast exchange (Supplementary Fig. 12) was assumed for all steps except those involving substrate oxidation or FAD and O_2_ reduction. For wild-type TbQSOX under the conditions of our experiments, FAD reduction is much slower than O_2_ reduction^12^, and these steps were represented by a single rate constant to make the model tractable. Expressions were derived for overall turnover and the ratio of closed to open molecules (see Methods). Data were fitted globally to the model (Fig. 4b), yielding an excellent fit and providing values for kinetic constants and ratios of species (Supplementary Table 2 and Supplementary Methods). While the rate constants obtained were consistent with values previously measured in rapid-reaction studies^12^, the relative concentrations of various conformational and chemical states constitute new information. Remarkably, in contrast to the greater than 90% of molecules open in chemical state A, only about 1% of the molecules in chemical state D were calculated to be open according to the ratio of D_cl_ to D_op_ obtained from the fits, suggesting that reduction of the Erv domain substantially affects the TbQSOX conformational energy landscape. Regarding the distribution among various reduced states in the cycling enzyme (Fig. 4c, Supplementary Fig. 13), D_cl_ was found to be about twice as populated as B, and very little C was calculated to exist. In summary, the novel outcome of this analysis is the steady-state distribution of molecules among the possible chemical and conformational options, as a function of substrate concentration.

### TbQSOX conformation gates alternative enzymatic sub-cycles

Application of the expanded kinetic model explains the origin of the two phases in TbQSOX smFRET and turnover. At low substrate concentrations, the rate-limiting step is insertion of electrons into the Trx active site (*k*_1_). Once the Trx domain is reduced (state B), the enzyme continues through state C to D_cl_. The increase in closed species as the substrate concentration increases in the first phase reflects this shift from A to D_cl_ (Fig. 4c). The preferential population of D_cl_ over D_op_ restricts the possibility of a second round of Trx reduction, but as the substrate concentration increases, reduction eventually outpaces other processes and generates four-electron reduced states. Thus, at high substrate concentrations the D→F→G→B sub-cycle competes with the standard D→E→A electron-transfer route. The decrease in closed species in the second phase reflects the shift from D and E to the obligate open species F and G.

In terms of turnover, increasing the number of molecules poised at the rate-limiting step (D_cl_, D_op_ and F) at the expense of molecules distributed among other states (B and C) results in rate enhancement. High DTT concentrations deplete states B, C, D_op_, and D_cl_ in favour of the open state F (Fig. 4c). Therefore, populating state F at high substrate concentrations simultaneously promotes open conformations and flux through the rate-limiting step.

**Figure 5:**
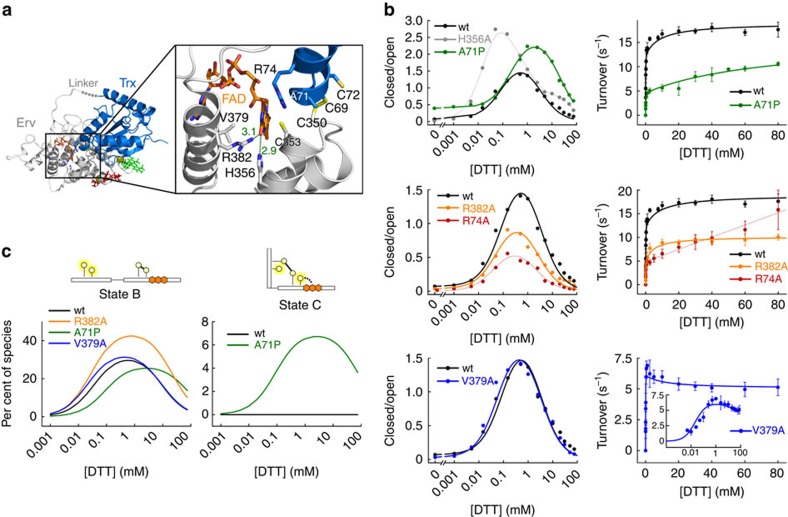
TbQSOX mutants test expanded kinetic scheme. (**a**) Close-up on TbQSOX interdomain interaction (PDB ID: 3QD9). The Trx domain is marine blue and the Erv domain is grey. Side chains of cysteines in both active sites and residues that were mutated are shown as sticks and labelled. Green numbers depict distances between atoms in Å. (**b**) Comparison of TbQSOX mutants in smFRET and oxygen consumption assays. Error bars represent s.d. of the mean of three measurements. With the exception of H356A and R74A, which are traced with dashed curves, the smFRET and turnover data are displayed with global fits to equations (4) and (5), respectively, derived from the TbQSOX model (see Methods). A global fit for R74A up to 2.5 mM DTT is presented in Supplementary Fig. 15. His356Ala showed no activity in oxygen consumption assays (Supplementary Fig. 14). (**c**) Steady-state levels of key TbQSOX species as a function of substrate concentration for various mutants compared to wild type.

Since state D_op_ is the junction between competing electron transfer routes, altering the rate constant for reducing D_op_ at the Trx domain (*k*_1_) or for transferring electrons further to FAD (*k*_3_′) and O_2_ (*k*_3_″) should affect the substrate concentration required to enter the four-electron cycle. TbQSOX mutants were produced to probe this branch-point and other aspects of the model, and smFRET and turnover data were collected for each mutant across the range of substrate concentrations. Two TbQSOX mutants, Ala71Pro and His356Ala (Fig. 5a), showed major changes in the maximum amount of closed conformer and in the substrate concentration at which this maximum occurred.

The mutant Ala71Pro populated closed states to a greater extent than wild type, and higher DTT concentrations were required to deplete these states (Fig. 5b). This mutant, which was produced previously and analysed using other techniques^18^, was reported to have a less oxidizing Trx active site than wild type, to prolong the existence of the interdomain electron-transfer intermediate, and to exhibit decreased turnover^18^. Quantitative analysis of our data according to the expanded TbQSOX model confirmed that steady-state levels of state C are higher in Ala71Pro than in wild-type TbQSOX (Fig. 5c, Supplementary Table 2), accounting for much of the excess closed conformer observed by smFRET for this mutant. Furthermore, the rate constant for Trx active-site reduction (*k*_1_) obtained from fits to the model was lower than for wild type (Supplementary Table 2), consistent with the decreased capacity of this mutant to accept electrons^18^. The lower rate constant for Trx reduction compromises the transition from D_op_ to F relative to the competing processes of back reaction toward state C and electron expulsion from the Erv domain, such that higher DTT concentrations are required to compensate. This compensation by increased substrate is evident from the shift of the closed species peak towards higher DTT concentrations in the smFRET results for Ala71Pro.

The second mutant, His356Ala, also showed greater accumulation of closed molecules, but unlike Ala71Pro, it exhibited this phenomenon at lower DTT concentrations than wild type (Fig. 5b). Also unlike Ala71Pro, which retained moderate activity, His356Ala was completely inactive (Supplementary Fig. 14). His356 normally makes a hydrogen bond to the FAD (Fig. 5a), and mutation of the comparable residue in other oxidases decreased the rate of electron transfer to the flavin^19^. In our studies, His356Ala was capable of interdomain electron transfer, as indicated by the shift to closed species on addition of low substrate concentrations. The presence of the second phase in the smFRET data further demonstrated that the His356Ala mutant does not become trapped in a closed state but likely proceeds to D_op_ and F by way of D_cl_. With little or no competition in His356Ala from the D→E→A route, relatively low DTT concentrations were apparently sufficient to deplete D_op_ by conversion to F. The Ala71Pro and His356Ala mutations both perturbed processes impacting D_op_, decreasing or increasing, respectively, the flux towards four-electron reduced species. These mutants thus emphasize how conformational exchange at the state D junction controls trapping of open species and produces the TbQSOX biphasic behaviour.

### Closed states guard the alternative electron entry port

In contrast to the greater accumulation of closed molecules in the mutants described above, less build-up of closed species was observed for two other TbQSOX mutants, Arg74Ala and Arg382Ala (Fig. 5b). The catalytic activities of both arginine mutants were also impaired (Fig. 5b). According to analysis based on the kinetic model (Fig. 5c, Supplementary Table 2 and Supplementary Fig. 15), cycling versions of both arginine mutants, particularly Arg74Ala, populate state B to a greater extent than wild-type TbQSOX. The relative accumulation of the arginine mutants in state B, an open state immediately upstream of two-electron-reduced closed states, suggests that these arginines have a role in assuming the closed conformation. A positive electrostatic environment contributed by Arg74 and Arg382 appears to promote electron relay to the Erv domain, presumably by stabilizing closed states with a negatively charged, reduced Erv domain.

By considering the locations of the two mutated arginines and their differential effects on enzyme turnover, further insight is gained into the TbQSOX kinetic mechanism and the role of protein conformation in controlling electron flow. Arg382 is in the Erv domain and located within hydrogen-bonding distance of the FAD. In this position, Arg382 is likely to promote electron transfer to the FAD (*k*_2_′ and *k*_3_′), the rate-limiting step of the TbQSOX catalytic cycle. Hence, Arg382Ala activity plateaued at a level lower than the wild-type enzyme (Fig. 5b). The other arginine, Arg74, is located in the Trx domain and interacts with the Erv domain active site only in the closed conformation. Arg74Ala reached about 30% of the wild-type activity at 1 mM DTT, but activity continued to increase linearly at higher DTT concentrations, approaching levels seen for wild-type TbQSOX (Fig. 5b). This behaviour can be explained by impaired flux through state C, which is bypassed at high DTT concentrations by direct reduction of the Erv domain (B→F), as demonstrated by the activity of the Trx AXXA mutant (Supplementary Fig. 9) and other mutants with defects in interdomain electron transfer^18^. In wild-type TbQSOX, this bypass route is negligible because electrons are captured and transferred efficiently by the Trx domain. By decreasing flux through the B→C→D_cl_ route and increasing flux through the B→F route, the Arg74Ala mutant revealed that closed enzyme states control not only the accessibility and reactivity of the Trx domain but also the possibility of direct reduction of the Erv domain by substrate. TbQSOX closure thus further suppresses electron entry into the system through the Erv domain, below the low levels expected based on the redox potential of this domain^18^.

### Conformational modulation of oxygen reduction

In the mutant Val379Ala (Fig. 5a), the fraction of closed molecules was indistinguishable from wild type across the entire DTT concentration range (Fig. 5b), but this mutant displayed an astounding inversion of the enhanced catalysis at high substrate seen for the wild-type enzyme (Fig. 5b). This apparent substrate inhibition can be explained by considering the contribution of enzyme conformation to yet another step in catalysis. Val379 lines the O_2_ route towards the FAD, and an analogous mutation in another flavoenzyme slowed oxidation of reduced flavin^19^. Perturbed TbQSOX oxidation could cause O_2_ reduction (*k*_2_″ and *k*_3_″) to become rate limiting (Fig. 4a). If a four-electron reduced state, an open state, dominates at higher DTT concentrations as expected, then the decreased activity seen at high substrate concentrations in Val379Ala implies that TbQSOX oxidation is slower in the open conformation than in the closed one. Indeed, fitting the model to the Val379Ala smFRET and turnover data yielded rate constants for FAD and O_2_ reduction differing by a factor of two depending on whether oxidation occurred from the closed conformer (*k*_2_) or the open conformer (*k*_3_) (Supplementary Table 2). The reader is reminded that the kinetic model developed for TbQSOX conflated the steps of electron transfer to the FAD and from FAD to O_2_ (Fig. 4a). The first of these steps is normally rate limiting, as the second occurs at least an order of magnitude faster^12^. In Val379Ala, the rate of electron transfer to O_2_ is expected to be depressed, such that this step may contribute to limiting the rate of turnover. In wild-type TbQSOX, any difference in oxidation rate for open versus closed forms would be masked by the slower upstream step of FAD reduction. Consequently, rates of Erv oxidation of open and closed molecules obtained from the fits for wild-type TbQSOX were identical (Supplementary Table 2). The Val379Ala mutant thereby exposes modulation of oxygen reduction as an additional effect of conformation on the TbQSOX cycle.

Altogether, this series of mutants probed a variety of transitions between enzyme states in the TbQSOX reaction scheme. The appropriate correspondence between the sites and roles of the mutated residues, the steady-state distribution of conformational species, and the catalytic activities of the mutants support the expanded TbQSOX scheme as a description of electron transfer pathways within the cycling enzyme.

## Discussion

The relationship between structural fluctuations in enzymes and catalysis of chemical reactions is under continuing discussion^20^,^21^,^22^,^23^,^24^,^25^. Although the general significance of conformational dynamics is by now well-established, much less developed is the employment of conformational exchange to elucidate enzyme mechanisms quantitatively. We demonstrate herein that conformational changes play a major role in the thiol- and flavin-based electron-transfer mechanism of the enzyme TbQSOX, and that these transitions can be incorporated into a coherent scheme coupling conformation and chemistry. Single-molecule FRET revealed that the steps in the TbQSOX electron relay are partitioned between two conformational ensembles of the enzyme. By recording the TbQSOX conformational distribution as a function of substrate concentration, we could extract information on chemical steps, since the conformational data provided tight constraints on the mechanistic model. At key junctions, conformation was found to gate the entrance of the enzyme into alternative sub-cycles or to partition the enzyme population into states with different reactivities.

By using smFRET to circumvent the relative spectroscopic silence of disulfide bond rearrangements, a number of key insights regarding TbQSOX were made. The two most important findings from this study were a pre-existing equilibrium in the absence of substrate between distinct conformational ensembles, ‘open' and ‘closed,' and the tuning of the occupancy of these ensembles by substrate concentration. The first of these observations indicated that the closed population is not restricted to the electron-transfer intermediate containing a disulfide bond between the two TbQSOX modules, pointing to the importance of including additional closed states in the kinetic model describing the dependency of TbQSOX conformation and activity on substrate concentration.

A third insight fostered by smFRET was the placement of four-electron reduced species in the TbQSOX mechanism. Over-reduced species were previously proposed for TbQSOX^12^ but were not observed or incorporated into earlier kinetic models. Indications of an alternative cycle in TbQSOX could be detected in bulk enzyme assays presented herein, but smFRET experiments exposed it conspicuously. Although the four-electron cycle predominates only at high substrate concentrations, its presence placed tight constraints over the entire TbQSOX mechanism, helping to illuminate the distribution of enzyme species in low as well as high substrate concentration ranges. It should be noted that the four-electron cycle will come into play for any substrate under conditions in which its rate of reduction of the Trx domain competes effectively with electron expulsion from the Erv domain, taking into account that Trx domain reactivity is severely limited by conformational state, whereas Erv oxidation proceeds in both the closed and open conformations. On the basis of studies of bacterial oxidoreductases^26^, native protein substrates may in principle reduce the TbQSOX Trx domain at rates greater than those observed for DTT. In addition, low oxygen levels, as trypanosomes may experience in their life cycle^27^, would slow the oxidation of the bound flavin. Either of these physiological scenarios would pull the enzyme into the four-electron cycle.

In addition to revealing two conformational populations smFRET measurements provided information on the nature of each population. A change in FRET signal can arise from toggling between two distinct conformations or from a switch in the range of configurations accessible by a flexible molecule. TbQSOX appears to combine both scenarios, with the enzyme switching between a distinct conformational state and a state spanning a wide range of conformations due to the flexible tethering of the two TbQSOX modules. In support of a distinct closed conformation, different energy transfer efficiencies were observed in the closed species for each donor label position, and the FRET efficiencies corresponded to expectation^16^ based on the crystal structure of a TbQSOX mutant^9^ mimicking state C. In contrast, the transfer efficiency for all labelled variants in the open state clustered around the value of 0.3, inconsistent with the enzyme merely switching to a new, distinct conformation with a different orientation between the two modules. Instead, the open state appears to represent tumbling of the two modules relative to one another on time scales faster than diffusion through the laser beam, such that the observed FRET efficiency is an average over the distribution. Functionally, the balance favouring the low FRET efficiency population in resting TbQSOX is consistent with the presumption that the enzyme should be open upon first encounter with substrate. A flexible open state in QSOX enzymes, which are localized to environments (that is, the Golgi and extracellular matrix) where assembly of multiprotein complexes takes place, may facilitate interaction with large and diverse substrates.

Despite the bias towards the open conformer in resting TbQSOX, some closed species is nevertheless observed in the absence of electron relay through the enzyme. The existence of a detectable fraction of closed molecules in resting TbQSOX indicates that the enzyme is primed for closure even with no covalent connection between the active sites. The affinity between the TbQSOX modules is finely tuned so as to slightly favour open forms when necessary but to allow ready conversion to closed states as required for turnover. We found that mutations near the TbQSOX redox-active sites altered the distribution between open and closed forms of the resting enzyme to either direction. For example, the Arg74Ala mutant lacks some interdomain interactions present in wild-type TbQSOX and exhibits less of the closed conformer (Supplementary Table 2). In Ala71Pro, a greater amount of the closed conformer was observed. The ease with which minor perturbations alter the amount of the TbQSOX closed conformer has potential functional implications. One important question regarding the biological function of QSOX enzymes is whether they are catalytically active during transit through the ER and thereby impact oxidative balance in the early secretory pathway. According to our observations, a hypothetical low-affinity interaction between an ER protein and closed QSOX would be sufficient to stabilize QSOX in an inactive conformation until it is trafficked past the ER.

Intriguingly, a TbQSOX mutant that had no obvious effect on the distribution of conformers in the cycling enzyme demonstrated an additional role for conformation in TbQSOX catalysis, in this case in relation to the oxidizing (that is, O_2_) rather than the reducing substrate. Of all the TbQSOX mutants studied, Val379Ala exhibited the most striking and unexpected effect on enzyme turnover, showing a marked decrease at high substrate concentrations. No hint of such behaviour was observed previously for any QSOX variant, and no mention of any mechanistic feature that could be relevant to this behaviour had been made. Consequently, uncovering the origin of this phenomenon provided novel insights into TbQSOX and, more generally, into enzymes bearing multiple internal routes within their mechanisms. We ascribed the behaviour of Val379Ala to the role of the Trx domain in oxygen reduction. One may speculate that the Trx domain contributes to the electrostatic environment favouring O_2_ activation, stabilizes the oxygen channel, or otherwise promotes transfer of electrons from the FAD to O_2_. Interestingly, in choline oxidase and polyamine oxidase, retention of the positively charged product of the reductive half reaction in the active site is proposed to promote O_2_ activation for the oxidative half reaction^19^,^28^. In the sense that the TbQSOX Trx domain can be considered the substrate for oxidation by the Erv domain, the continued presence of the Trx domain in state E_cl_ may contribute to O_2_ activation in an analogous manner.

It is notable that the behaviour of wild-type and Val379Ala TbQSOX as a function of substrate concentration resembles the phenomena of substrate activation and inhibition. These phenomena are often assumed to be allosteric effects caused by substrate binding to a second site in an enzyme, altering the catalytic rate at the active site. In wild-type TbQSOX, a reducing substrate interacts with a single site, namely the Trx CXXC motif. It has been recognized for many decades, however, that substrate activation and inhibition can also arise from competition between alternate pathways in certain two-substrate enzymes^29^,^30^. We show here that even for an enzyme that interacts in a relative simple manner with a single reducing substrate at a single active site, diverse patterns of activity as a function of substrate concentration can be achieved by subsequent internal transitions within the protein, without invoking direct substrate binding at non-catalytic sites. With access via smFRET to a previously hidden region of the TbQSOX kinetic scheme, the origins of these patterns were quantitatively revealed. Remarkably, the smFRET measurements, while not reporting directly on electron transfer between the multiple redox-active sites of TbQSOX, provided insights into the interplay between enzyme states that differ in their chemistry (that is, location of electrons along the relay) as well as those that differ in conformation.

## Methods

### Production and labelling of TbQSOX

TbQSOX mutants were made by restriction-free cloning on the basis of the published TbQSOX expression plasmid^9^,^12^. Wild-type and mutant enzymes were produced in the Origami 2 (DE3) *E. coli* cell strain (Novagen) and purified as described^9^. Unlabelled proteins were exchanged by gel filtration into PBS in the last purification step. Proteins destined for labelling with only one fluorophore (for Förster distance calculation) were incubated with a threefold molar excess of either Alexa Fluor 594 C5 maleimide or Alexa Fluor 488 C5 maleimide (Invitrogen) for 30 min at room temperature. After labelling, proteins were applied to a HiLoad 16/60 Superdex 75 gel filtration column (GE Healthcare) in PBS to remove any excess dye. Proteins destined for double-labelling were first exchanged into 20 mM sodium phosphate buffer, pH 7.5. An equivalent molar amount of Alexa Fluor 488 C5 maleimide was added, and the first labelling reaction was allowed to proceed for 30 min at RT. The cysteine introduced at position 243 was poorly reactive with this negatively charged dye, most likely because position 243 is in a negatively charged electrostatic environment in the protein (Supplementary Fig. 1b). In contrast, the other cysteines introduced into TbQSOX, at each of the six positions, were readily labelled with the donor fluorophore. Protein labelled on one cysteine was isolated on a Mono Q 5/50 GL column (GE Healthcare) run in 20 mM sodium phosphate buffer, pH 7.5, and eluted with a NaCl gradient to 0.5 M over 100 min. Alexa Fluor 594 C5 maleimide, in the same amount used for donor labelling, was then added to label position 243. After 30 min at RT, double-labelled TbQSOX was purified on the Mono Q column with the same gradient as above. For storage, 10% glycerol was added to the protein stock solutions, which were then divided into aliquots, flash-frozen in liquid nitrogen, and placed at −80 °C.

### Verification of homogeneity in TbQSOX labelling

Double-labelled TbQSOX variants were incubated at room temperature for 15 min with various concentrations (100–0.4 μg μl^−1^) of trypsin (Sigma). The primary cleavage sites were in the flexible, interdomain linker, generating major fragments containing either the Trx or the Erv domain, identified by mass spectrometry fingerprinting (data not shown). After quenching the reaction with Laemmli sample buffer, the fragments were separated on an SDS polyacrylamide gel and read in a Fujifilm FLA 1500 laser scanner at two wavelengths, one compatible with the donor excitation wavelength (473-nm laser and 510-nm filter), and the other with the acceptor wavelength (532-nm laser and 575-nm filter). The full-length protein was observed in both channels (Fig. 2b, Supplementary Fig. 1a, yellow bands), whereas the Trx band was observed only when excited at a wavelength compatible with the donor label (green bands) and the Erv band only when excited at a wavelength compatible with the acceptor label (red bands). These results confirm that the double-labelled TbQSOX was uniform in its dye positions. This quality control was performed on all labelled TbQSOX variants and mutants.

### TbQSOX bulk turnover assays

TbQSOX activity was measured at 25 °C by monitoring oxygen consumption in a Clarke-type oxygen electrode (Hansatech Instruments Ltd). TbQSOX at 50 nM (or 100 nM when measuring activity of certain TbQSOX mutants) was assayed with various DTT concentrations. Reactions were initiated by injection of DTT into the reaction chamber and were repeated three times for each DTT concentration. Turnover numbers were calculated from slopes of oxygen depletion progress curves. Double-injection assays were performed and analysed as follows. According to the Michaelis–Menten model, an enzyme with a *K*_M_ of 65 μM is expected to function at 94% of *V*_max_ on 1 mM substrate. At increasing substrate concentrations, the ratio of the reaction rate to the rate at 1 mM substrate should plateau at 1.065. Oxygen consumption rates at various DTT concentrations were measured relative to the rate on 1 mM DTT by first supplying TbQSOX (50 nM) with 1 mM DTT, recording the oxygen consumption rate for 1 min, making a second injection of DTT (or only buffer) to bring the final DTT concentration to between 1 and 80 mM, and recording the new oxygen consumption rate. The ratio of the two rates for each sample, after correction for small volume changes, was then taken, and ratios were averaged for multiple measurements at the same final DTT concentration (*n*=3–6). Error bars represent s.d. of the ratios.

### Sample preparation for single-molecule experiments

Sample cells were prepared as described^31^ and washed with 20 mM sodium phosphate buffer, pH 7.5, 200 mM NaCl containing 0.01% Tween. Cells were then filled with a mixture of 25-pM-labelled TbQSOX and DTT at various concentrations. The cells were sealed with silicon grease to prevent water evaporation during measurement.

### smFRET experiments on freely diffusing molecules

Measurements were conducted on a custom-made single-molecule microscope for automated data collection^31^. The laser beam was focused 14 μm into the solution, and data were collected continuously at 22 °C for one hr before the sample was replaced. Several such data sets were collected for each TbQSOX variant and in each condition. Notably, taking into account the low TbQSOX concentration used in the experiment (25 pM) and TbQSOX maximal turnover (18 s^−1^=64,800 h^−1^), about 1.6 μM O_2_ would be consumed enzymatically per hour. Since the concentration of O_2_ in aqueous solution is ∼250 μM, in a typical hour-long smFRET experiment only about 6% of the O_2_ in the solution was expected to be consumed. Detection of photon bursts and their analysis were performed as described previously^32^.

### Data analysis of free diffusion experiments

FRET efficiencies calculated for all bursts in a certain measurement were binned into 49 bins. For D-116 and its mutants, FRET efficiencies larger than 0.7 were considered as ‘high FRET,' and FRET efficiencies below 0.7 (excluding FRET efficiencies below 0.1, which constitute the ‘donor only' peak) were considered as ‘low FRET' for the closed/open ratio calculations.

### Apparent distance calculations

Apparent distances between the dye reporters were calculated for the closed state based on FRET efficiencies according to the equation:


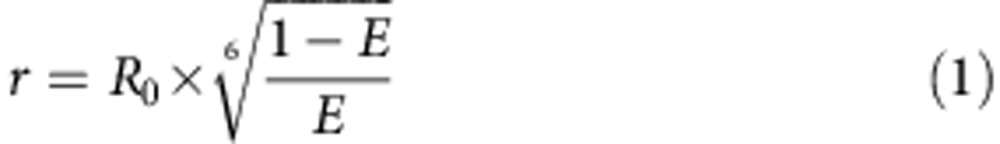


Where *r* is the distance between the dyes, *R*_*0*_ is the calculated Förster distance (see below), and *E* is the FRET efficiency value at the centre of the peak in the single-molecule FRET efficiency histograms corresponding to the closed conformation. Since the Förster distance of the dye pair may be sensitive to the dye's chemical environment, it was calculated for each donor position according to the equation:


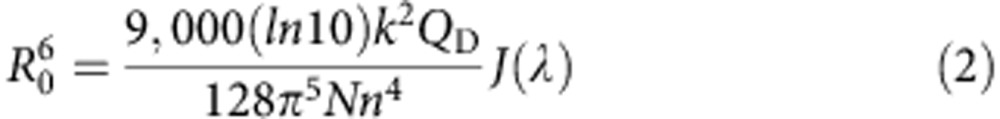


Where *k*^2^ is the orientation factor of the dyes taken to be 2/3, *N* is Avogadro's number, *n* is the buffer's refraction index measured to be 1.3355, *Q*_D_ is the quantum yield of the donor fluorophore, and *J*(*λ*) is the overlap between the donor emission spectrum and the acceptor absorbance spectrum.

The FRET efficiency values were corrected for dye quantum yields before distance calculations were performed. The donor quantum yield was measured in bulk fluorescence assays in a Fluorolog spectrofluorometer (Jobin–Yvon) for each donor position, in reference to the quantum yield of fluorescein (95% in 0.1 M NaOH). The emission spectra of TbQSOX labelled with only a donor at position 189, were collected at five concentrations, three times in each concentration, under the same excitation conditions (496 nm, 22 °C) in the buffer used for smFRET experiments. The quantum yield was found from the ratio between the dye's integrated emission spectrum and its absorbance at 496 nm. The emission spectra of all five other TbQSOX labelled variants were collected at two concentrations, and their quantum yields were measured similarly.

The overlap between the donor emission spectrum and the acceptor absorbance spectrum is defined as:


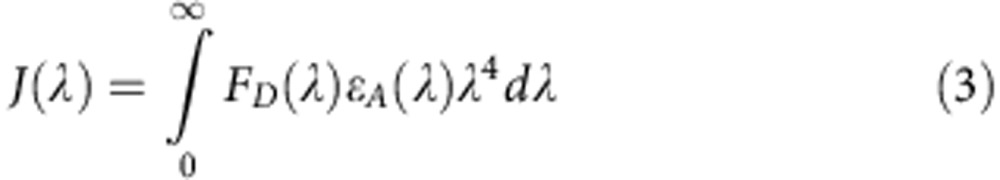


Where *F*_D_ (*λ*) is the normalized donor emission spectrum, and *ɛ*_A_ is the acceptor's absorbance spectrum, measured for TbQSOX labelled with an acceptor at position 243.

Calculated Förster distances and distances between donor and acceptor in the closed conformation according to smFRET experiments are presented in Supplementary Table 1.

### FRET-fluorescence correlation spectroscopy measurements

The same set-up was used as for freely diffusing molecules. Measurements were performed on 500 pM TbQSOX D-116, using a laser power of 50 μwatt. The donor–acceptor cross correlation function was calculated using the algorithm of Wahl *et al*.^33^

### Stopped-flow experiments

Measurements were conducted using an Applied Photophysics stopped-flow apparatus. Reactions were initiated by mixing equal volumes of 500 nM TbQSOX D-147 and various DTT concentrations at 25 °C. Donor fluorescence was filtered by a Semrock FF01-536/40 filter and monitored as readout of conformational change. Five to ten traces were collected for 0.5 s (using a split-time base of 0.05 and 0.5 s with 1,000 sampling points in each time interval) for each DTT concentration. Rate constants obtained from fitting the traces to a single exponential were averaged. Traces were collected in the absence of DTT as well, to verify that the observed decrease in donor fluorescence was due to TbQSOX activity.

### Model generation and analysis

Four-electron species, namely states F and G (Fig. 4a), were added to the previous TbQSOX model shown in Fig. 1. Each chemical species was assigned to a closed or open conformation, or both. State C has an interdomain disulfide and hence is covalently constrained to be closed. States F and G arise via other open states, so these chemical species originate in the open form. As F and G have two electrons on each domain, electrostatic repulsion may continue to favour the open conformation. State B similarly arises in the open state, because the Trx domain must be accessible to DTT in solution. Although closure of state B is a prerequisite for forming the interdomain disulfide and converting to state C, the closed B state was not explicitly incorporated into the model. The remaining species, namely A, D and E, were assigned both an open and a closed conformation.

The model for TbQSOX presented in this study was developed to account quantitatively for bulk turnover and single-molecule FRET data. To adequately represent the data, additional states, beyond those shown explicitly in a previous scheme^12^, were proposed to be kinetically relevant. However, the states presented in Fig. 4a and described above should not be considered as exhaustive. Other combinations of chemistry and conformation may exist.

Some steps in the TbQSOX model (Fig. 4a) were assumed to be reversible, whereas others were considered irreversible. Redox reactions in one domain were assigned the same rate constant whether the other domain was oxidized or reduced. Rate constant *k*_1_ represents oxidation of DTT at the Trx active site. Reduction of FAD and reduction of O_2_ were treated as a single reaction, which was assigned a different rate constant for closed species versus open species (*k*_2_ and *k*_3_, respectively). The model was solved under a steady state assumption. The ratios between the concentrations of certain species that exchange in both directions was taken to be constant, and were assigned accordingly: 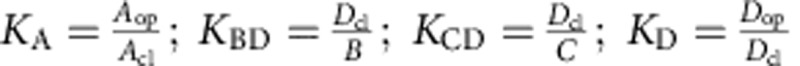.

*K*_*A*_ was extracted from single-molecule measurements in the absence of substrate, as the ratio between the closed and open populations (Fig. 2c), and input as a fixed value into the expressions. The ratio between closed and open species is provided by equation (4):


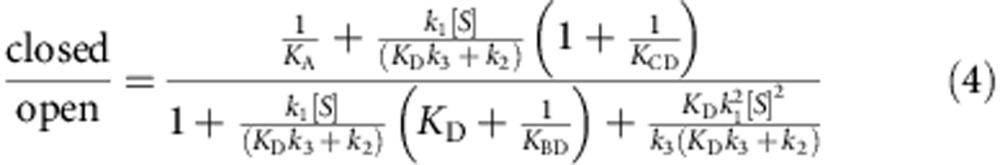


Equation (5) provides the turnover rate of the enzyme at substrate concentration [S], as measured by bulk oxygen consumption:


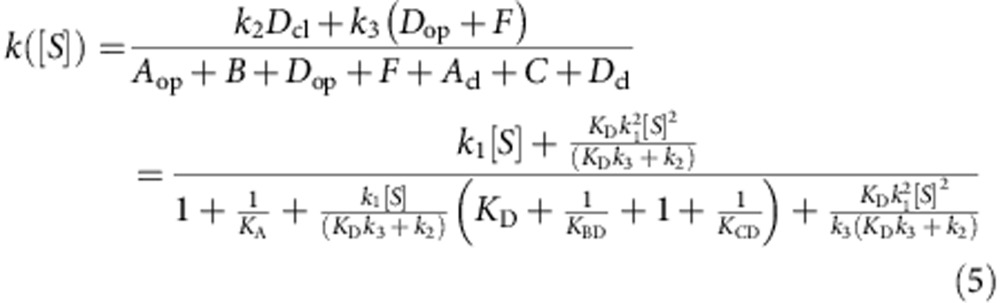


The closed/open ratio obtained from the smFRET measurements and the bulk turnover data for wild-type TbQSOX and for each mutant were fitted globally to equations (4) and (5), respectively, to obtain joint parameters (Figs 4b and 5b, Supplementary Table 2). The fraction of each enzyme species as a function of DTT concentration was calculated using these parameters (Fig. 4c).

Since the direct reduction of the Erv domain by substrate, which was not included in the kinetic model, becomes significant at DTT concentrations above 5 mM for Arg74Ala, the data for this mutant were fitted to equations (4) and (5) only up to 2.5 mM DTT (Supplementary Fig. 15).

## Additional information

**How to cite this article:** Grossman, I. *et al*. Single-molecule spectroscopy exposes hidden states in an enzymatic electron relay. *Nat. Commun.* 6:8624 doi: 10.1038/ncomms9624 (2015).

## Supplementary Material

Supplementary Information Supplementary Figures 1-15, Supplementary Tables 1-2, Supplementary Methods and Supplementary References
