# Supplementary material for: Single-molecule spectroscopy exposes hidden states in an enzymatic electron relay
Source: Nat Commun. 2015 Oct 15;6:8624. doi: 10.1038/ncomms9624 (PMC4634331; doi:10.1038/ncomms9624)
Supplement: Supplementary Information — Supplementary Figures 1-15, Supplementary Tables 1-2, Supplementary Methods and Supplementary References [file ncomms9624-s1.pdf]

Supplementary Figures

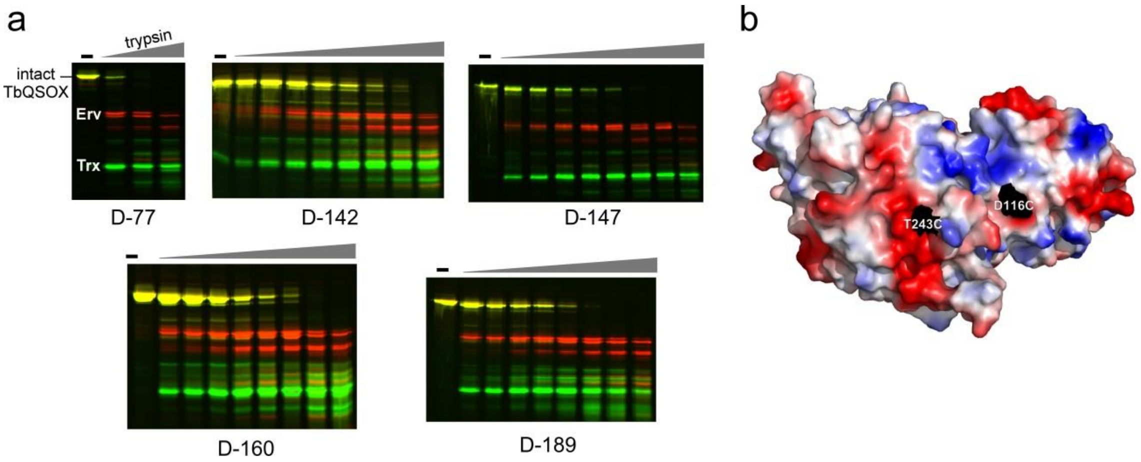

Supplementary Figure 1 | Labeling of TbQSOX variants. (**a**) Labeled TbQSOX variants were subjected to increasing concentrations of trypsin (represented by gray wedges), which cleaves preferentially between the Trx and Erv domains. The proteolysis products were separated by SDS-PAGE, and gels were read in a laser scanner with excitation at 473 nm (green bands), and at 532 nm (red bands), as described in the Methods section. Bands emitting in both channels appear yellow. Only full-length labeled TbQSOX is yellow, indicating that each distinct proteolytic product contains either a donor or an acceptor fluorophore and not a mixture. The donor fluorophore is on the Trx domain and the acceptor fluorophore is on the Erv domain in each variant. (**b**) The negatively charged electrostatic environment of position T243C likely explains its poor reactivity towards labeling with Alexa 488 C5 maleimide, as demonstrated by an electrostatic surface view of TbQSOX (PDB ID: 3QD9), with negative charge in red and positive charge in blue. For comparison, a position (D116C) that readily accepted the donor label is shown.

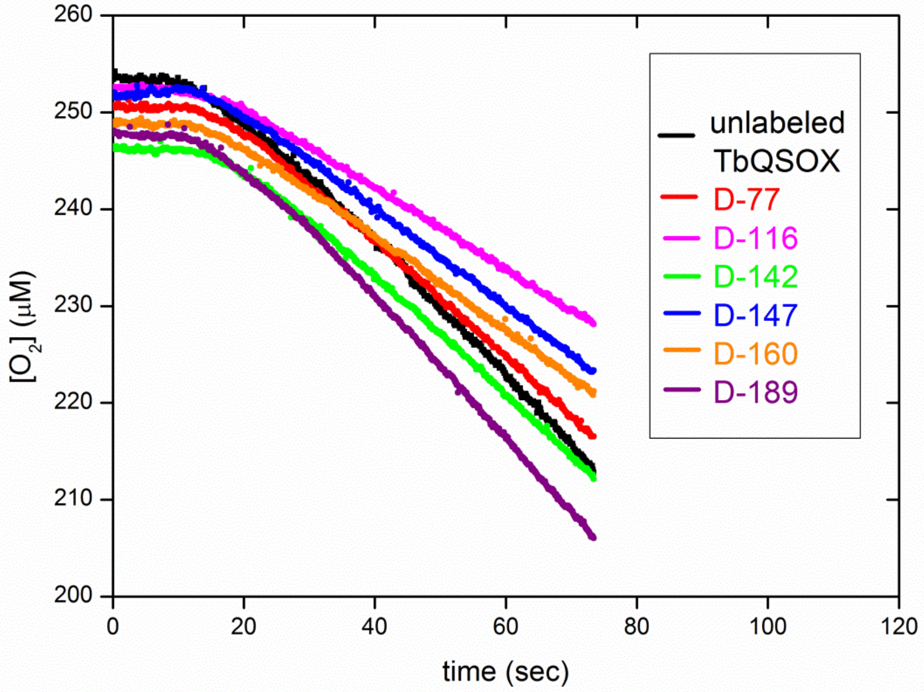

Supplementary Figure 2 | Activity of labeled TbQSOX variants. DTT was injected at t = ~15 seconds to a final concentration of 1 mM into 50 nM enzyme solutions. The resulting decrease in dissolved oxygen concentration is an indication of enzymatic oxidation of DTT. Steady state oxygen consumption rates of all labeled variants were at least 60% that of unlabeled wild-type enzyme.

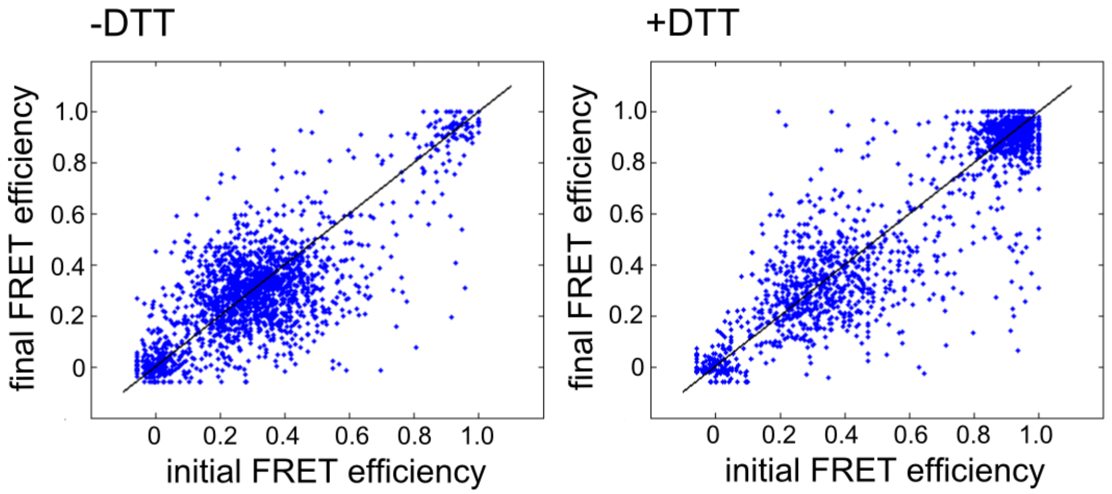

Supplementary Figure 3 | Transition rate between conformations is slow relative to burst duration. The average burst duration was measured to be 800 +/- 2 μsec (standard error of the mean). Transition diagrams are shown for 3000 bursts collected from D-116 in the absence of substrate (left) and presence of 1 mM DTT (right). Each burst was divided into two sub-bursts equal in their duration, and FRET efficiencies were calculated for each sub-burst. Each point in the transition diagram associates the FRET efficiency of a sub-burst with its complementary sub-burst. Few transitions between high and low FRET efficiency values were observed during bursts, as most points cluster around the diagonal. A photon-by-photon maximum likelihood analysis of bursts[1] confirmed that the dwell times in the open and closed states were comparable with the typical burst duration.

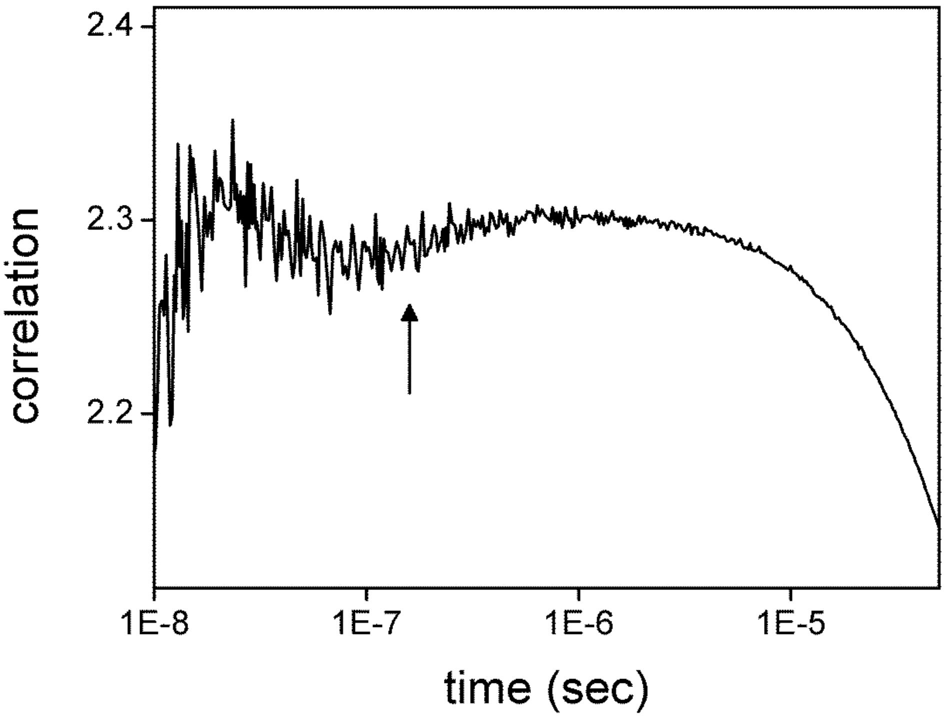

Supplementary Figure 4 | FRET-FCS measurements reveal internal motions in the sub-microsecond timescale. Donor-acceptor cross-correlation measured for TbQSOX D-116. Several processes affect the correlation function. The positive correlation in the sub-millisecond timescale is due to molecular diffusion. The anti-correlation in the nanosecond timescale is due to photon anti-bunching. The anti-correlation indicated with an arrow is a result of fluctuations in FRET efficiency on a timescale of sub-microseconds. Since this experiment was done on oxidized TbQSOX, which is found mainly in the open conformation, the fluctuations on this timescale likely represent fluctuations within the energy well corresponding to the open conformation, *i.e.*, tumbling of the two modules tethered by the interdomain linker relative to each other. The sub-microsecond timescale is much shorter than the average time that each molecule spends at the laser beam, so the fluctuations observed in the cross-correlation function are averaged in the histogram (Fig. 2c) to yield one low-FRET efficiency peak, observed for all labeled TbQSOX variants, regardless of the donor position.

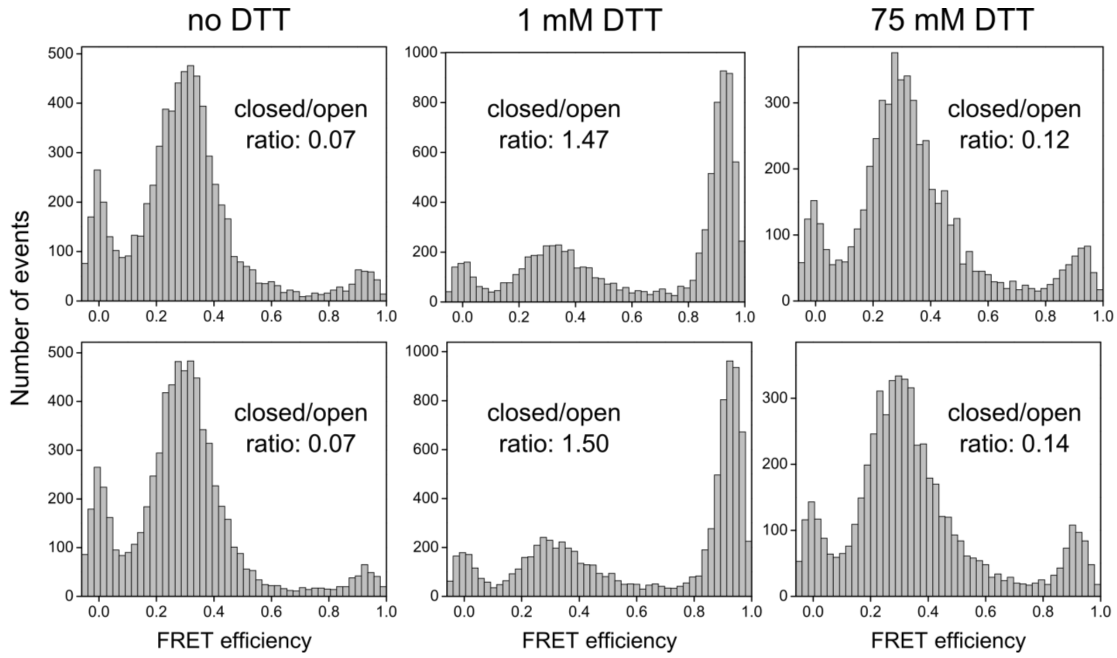

Supplementary Figure 5 | Single-molecule FRET measurements were performed under steady-state conditions. Histograms are shown for smFRET measurements of TbQSOX D-116 with various DTT concentrations (designated above the histograms). The data from the first 30 minutes of the measurement are presented in the upper panels and from the next 30 minutes in the lower panels. The calculated closed/open ratios using data from each histogram are shown. There is no appreciable difference between the histograms representing non-overlapping time windows of the measurements, nor is there a change in the closed/open ratio, indicating that the measurements were performed under steady-state conditions.

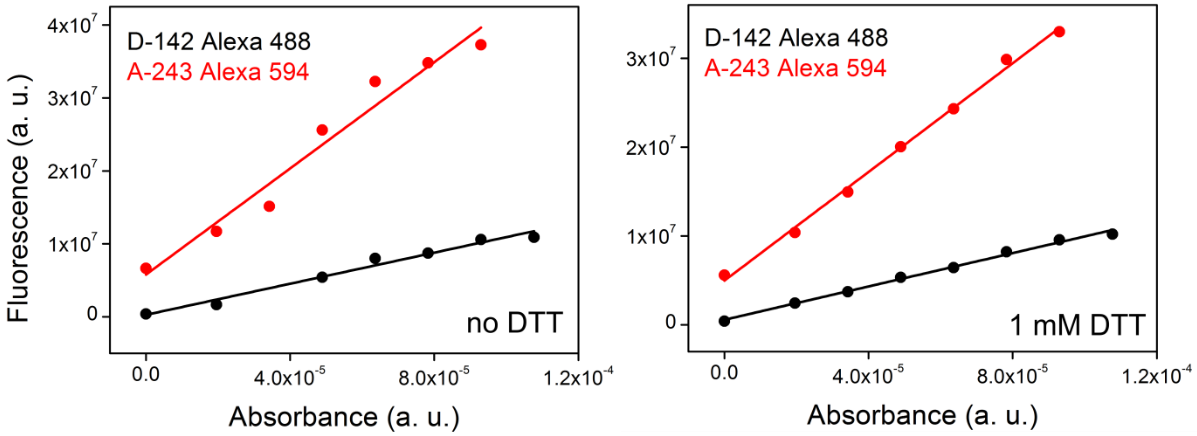

Supplementary Figure 6 | DTT does not affect the photo-physical properties of the fluorescent dyes. Quantum yield measurements for wild-type TbQSOX labeled only with Alexa 488 dye at position 142 (D-142 Alexa 488) or with Alexa 594 dye at position 243 (A-243 Alexa 594). Seven different dilutions of the protein were excited in the absence (left) or presence of 1 mM DTT (right), and fluorescence was measured. Data were fit linearly, and quantum yields for each dye were obtained from the slopes. No significant difference was detected when comparing the quantum yields of both dyes in the absence and presence of 1 mM DTT.

$\frac{Q_D}{Q_A}$ (- DTT)	$\frac{Q_D}{Q_A}$ (+ DTT)		$\frac{Q_{D(-DTT)}}{Q_{D(+DTT)}}$	$\frac{Q_{A(-DTT)}}{Q_{A(+DTT)}}$
0.72±0.05	0.68±0.02		1.13±0.08	1.2±0.1

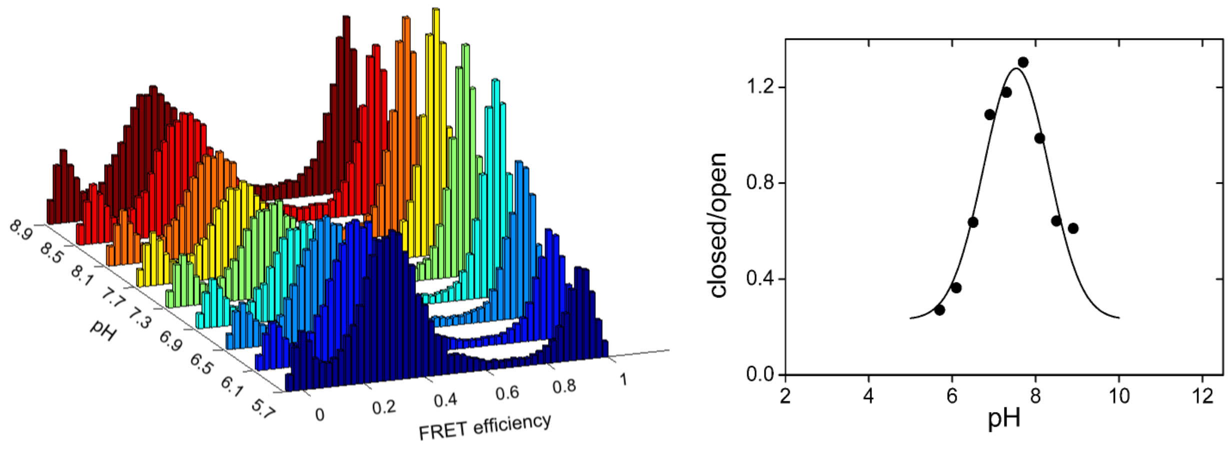

Supplementary Figure 7 | pH profile of TbQSOX turnover reflects partitioning to the closed state. Single-molecule experiments were performed using D-116 and 1 mM DTT. The left panel shows FRET efficiency histograms collected at various pH values. Experiments were conducted in 200 nM NaCl and either 50 mM sodium phosphate (pH 5.7-7.7) or 50 mM Tris (pH 8.1-8.9). The plot to the right, showing the ratios of closed to open populations calculated from the histograms, is comparable to the pH profile of TbQSOX activity[2].

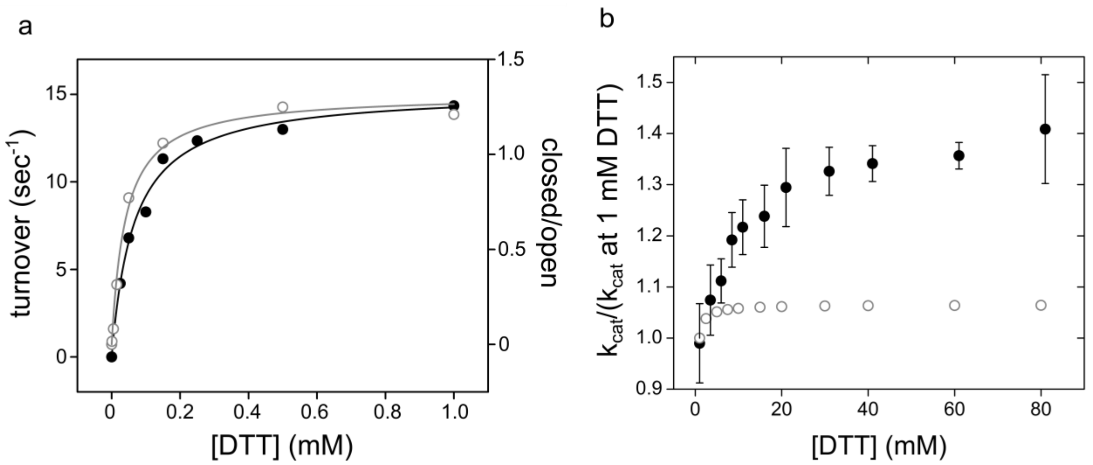

Supplementary Figure 8|Comparison of substrate concentration dependence of TbQSOX turnover and conformational distribution, and deviation from the Michaelis-Menten model at high substrate concentrations. (**a**) Michaelis-Menten fit to TbQSOX bulk turnover experiment (filled circles) and smFRET populations (open circles), from 0 to 1 mM DTT. (**b**) Excess velocity of TbQSOX turnover (filled circles) compared to Michaelis-Menten expectation based on the fit in panel **a** (open circles). To improve the precision of relative rate measurements, low and high DTT concentrations were injected successively into the same enzyme solution (see Methods). The results confirmed deviation from the Michaelis-Menten model with a K_M of 65 μM.

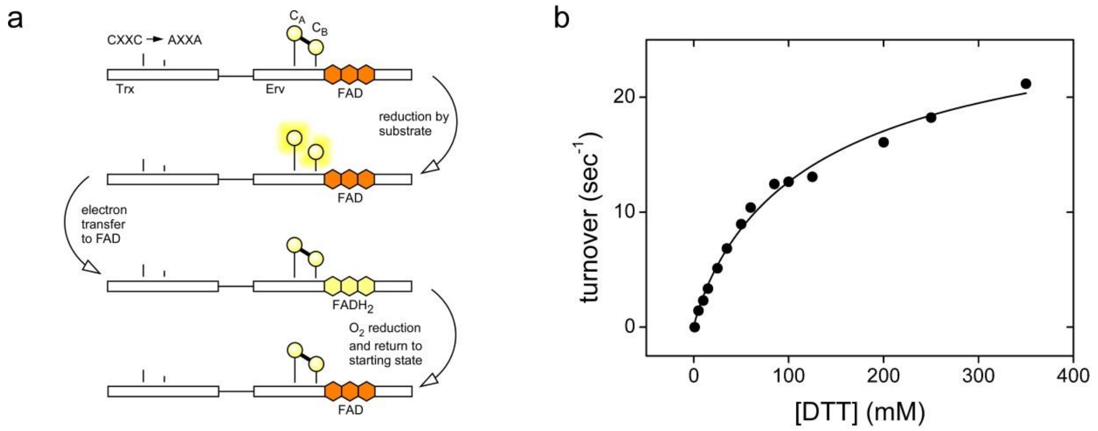

Supplementary Figure 9 | Testing the "second port" hypothesis by measuring the apparent K_m of the Erv CXXC motif. The relatively low apparent K_M (~65 µM) of the TbQSOX Trx domain for DTT implies that the primary entry port for electrons in QSOX is the Trx disulfide. With increasing DTT concentrations, direct reduction of the Erv domain CXXC motif by DTT might be expected to compete with nucleophilic attack by a reduced Trx domain and cause the shift to the open conformer at high DTT concentrations. (**a**) The Trx AXXA mutant is incapable of accepting electrons at the normal entry point into the enzyme and consequently incapable of shuttling electrons between domains. (**b**) In bulk enzyme assays, the AXXA mutant was found to have an apparent K_M of 100 mM, more than an order of magnitude higher than the DTT concentrations that deplete the closed conformation in the single-molecule experiments. Moreover, turnover of the AXXA mutant continued to rise as the DTT concentration was increased up to 80 mM DTT and beyond, whereas wild-type activity plateaued already at 20 mM DTT. Therefore, reduction of TbQSOX by DTT at the Erv domain is unlikely to explain the biphasic behavior of TbQSOX in smFRET and turnover experiments as a function of DTT concentration. Consequently, the B → F transition was not incorporated into the expanded TbQSOX kinetic model.

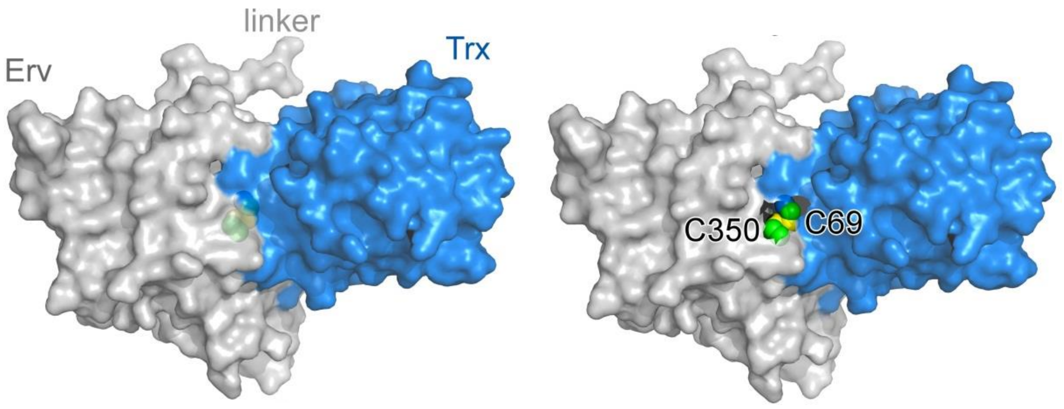

Supplementary Figure 10 |Exploring the "attack on the interdomain disulfide" hypothesis by evaluation of the TbQSOX closed conformer structure. If TbQSOX were to populate the interdomain disulfide intermediate during steady-state turnover at low and intermediate DTT concentrations, DTT at high concentrations might directly reduce the interdomain disulfide, enhancing turnover and liberating the enzyme to sample open configurations. Examination of the TbQSOX interdomain electron-transfer mimic structure[3], however, reveals that the interdomain disulfide is shielded from solvent, likely making it unreactive with exogenous substrate. In the left panel, the interdomain mixed disulfide is barely visible through the semi-transparent surface. In the right panel, a window has been cut in the surface to reveal the buried Cys69-Cys350 disulfide. On the basis of this analysis, state C was not considered to be reactive with DTT in the expanded TbQSOX kinetic model.

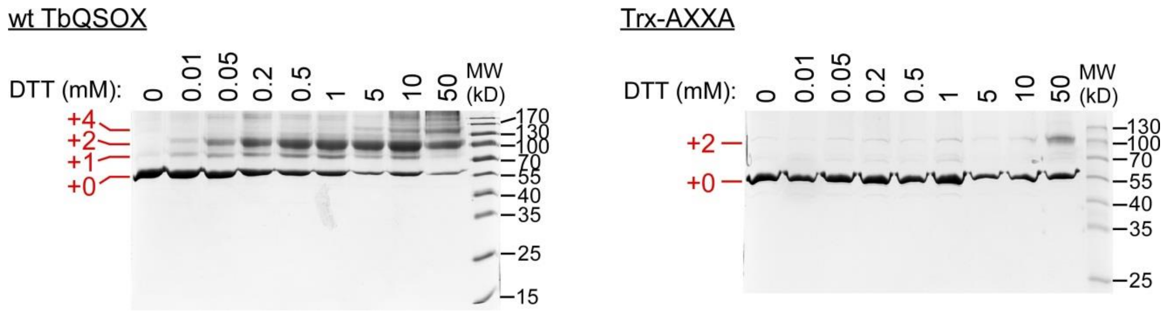

Supplementary Figure 11 | Testing the "regulatory disulfide" hypothesis by counting reduced TbQSOX cysteines at various substrate concentrations. Reduction of a regulatory disulfide is a possible explanation for increased enzymatic activity and the drop in closed states in high DTT concentrations. For example, if resolution of the interdomain disulfide were the rate-limiting step in the TbQSOX cycle, and if reduction of a distinct regulatory disulfide were to expedite resolution of the interdomain disulfide, then reduction of the regulatory disulfide at high DTT concentrations might promote enzyme opening and enhance catalysis. To detect reduced cysteines in cycling TbQSOX, we quenched reactions by rapidly lowering the pH and subsequently modified free thiols with maleimide-functionalized polyethylene glycol of molecular weight 5000 Da (mal-PEG), which does not react with cysteines in disulfide bonds. This experiment revealed the appearance of two reactive cysteines as the DTT concentration increased past the Km for the Trx domain, but no additional cysteines were reduced as the DTT concentration reached levels associated with the drop in the population of closed conformer (compare 1 and 5 mM DTT lanes in wild type). To confirm this observation, the Trx AXXA mutant was subjected to the same treatment. This enzyme variant, which possesses all possible regulatory disulfides, did not exhibit reactive cysteines below 50 mM DTT. Apparently, all disulfides in QSOX, with the exception of the Trx CXXC motif, are highly resistant to reduction and are not likely to be involved in events occurring with a DTT concentration midpoint in the low millimolar range. As a result of this observation, no disulfides other than the Trx and Erv CXXC motifs were considered to play a role in the expanded TbQSOX kinetic model.

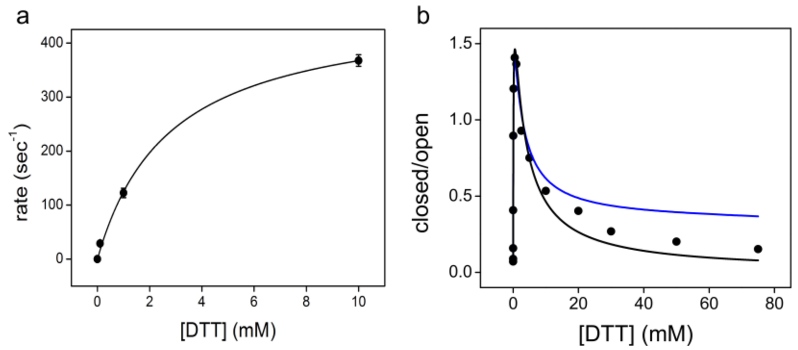

Supplementary Fig. 12 | Analysis of the fast conformational exchange assumption. Previous rapid reaction studies[2] showed that inter-domain electron transfer, which requires a conformational change, is faster than oxidation of the Erv module (280 sec^{-1} vs. ~19 sec^{-1}). Here we explore the assumption of fast conformational changes in the TbQSOX model using stopped flow measurements of conformational redistribution and simulations varying the rates of conformational exchange between D_{cl} and D_{op}. (**a**) Stopped flow experiments were performed on 250 nM TbQSOX D-147 mixed with various DTT concentrations. Donor fluorescence was monitored as readout of conformational change. Traces were fitted to single exponentials, yielding increasing rate constants for conformational changes with increasing DTT concentrations up to at least 10 mM. Thus, k_1, and not conformational changes, remains rate-limiting up to at least 10 mM. (**b**) Black symbols represent the experimental data presented in Fig. 3b and 4b. The black line is the global fit as shown in Fig. 4b. The blue line is a simulation of the model with the same parameters obtained from the fit, only instead of equilibrium constants for reversible reactions, two rate constants were used for the backward and forward reactions, with the ratio between them fixed and identical to the one obtained from the global fit. The conformational exchange between D_{op} and D_{cl} was set to be slow: $K_D = \frac{50\ sec^{-1}}{5{,}000\ sec^{-1}}$, leading to deviation from the model, especially at high DTT concentrations. The actual experimental data is more similar to the model, which assumes fast exchange, than to the slower exchange simulation. However, some deviation can also be seen for the experimental data at high DTT concentrations. The mutant that fits the model best at high DTT concentrations (Fig. 5b) is the Ala71Pro mutant, which has the lowest k_1 value (Supplementary Table 2). Evidently, the fast conformational exchange assumption is suitable over most of the DTT concentration range, but at high DTT it begins to lose its validity, unless k_1 is depressed.

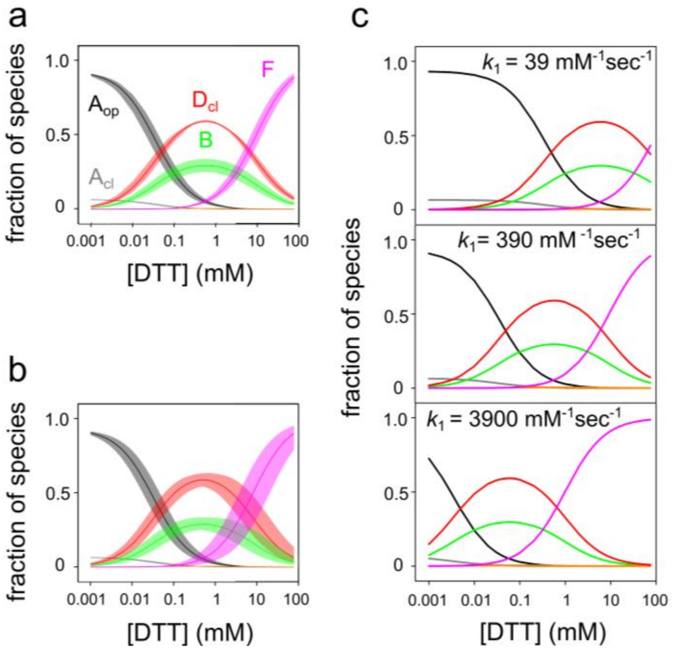

Supplementary Fig. 13 | Simulations of TbQSOX species distributions with varying parameter values. (**a**) The steady-state distribution in Fig. 4c (shown here in lines) was recalculated using the boundary values of the 95% confidence range for each parameter (reported in Supplementary Table 2), while allowing the other parameters to change accordingly. For each species, the two distributions that deviated the most from the one shown in Fig. 4c defined the error band for the distribution of that species (shown as a shaded background). The errors in parameters do not greatly affect the TbQSOX species distribution. (**b**) Same as in **a**, only the error bands were calculated by sampling randomly a thousand values of each parameter within the 95% confidence range. As opposed to the simulation in **a**, all parameters were varied independently of each other, thus generating 1000 DTT-dependency curves for each species. The minimal and maximal values for each enzyme state at each DTT concentration are shown as error bands. As expected, the variation of all parameters within their confidence increases the error bands compared to **a,** but still the TbQSOX species distribution supports all conclusions drawn in this work. (**c**) The k_1 rate constant was varied by an order of magnitude in either direction from the value obtained from the global fitting (middle), to simulate the effects of substrates that react with TbQSOX at different rates. The main effects of varying k_1 are on the substrate concentration at which the maximum amount of closed state is observed, and on the substrate concentration at which four-electron reduced species are observed. Other features of the population distributions remain the same.

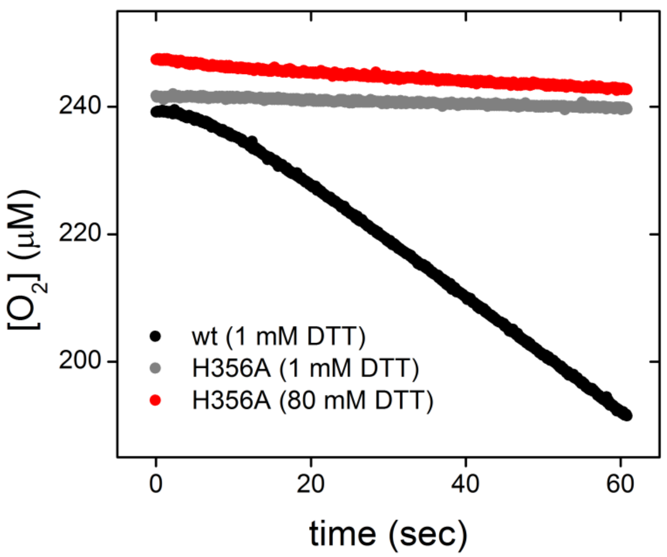

Supplementary Figure 14 |TbQSOX His356Ala is not active at any DTT concentration tested. DTT was injected to a final concentration of 1 mM into 50 nM enzyme solutions of wild-type TbQSOX. The resulting decrease in dissolved oxygen concentration is an indication of enzymatic oxidation of DTT. When DTT was injected to a final concentration of 1 mM or 80 mM into 200 nM enzyme solutions of His356Ala, no decrease in dissolved oxygen concentration was observed, indicating that this mutant is inactive.

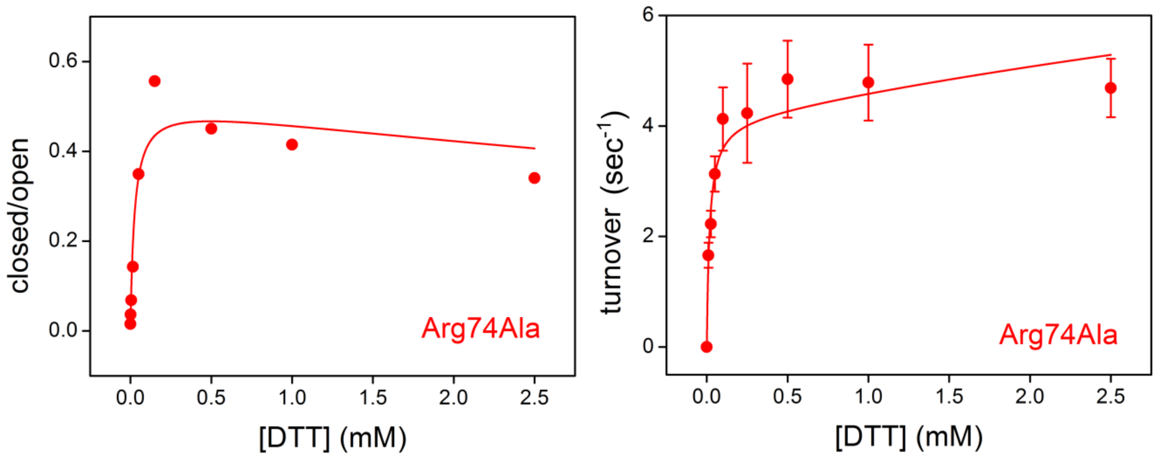

Supplementary Figure 15 |Global fit of enhanced model to smFRET and bulk turnover data for Arg74Ala. (Left) Ratios between closed and open populations as presented in Fig. 5b. (Right) Bulk turnover data as presented in Fig. 5b. The two data sets were fitted globally to the model from 0 to 2.5 mM DTT. According to the fit, the ratio between state D_{cl} and B (K_{BD}) in steady state is 0.52 (Supplementary Table 2). Due to the small number of data points, k_2 and k_3 were set equal in this fit. Allowing k_2 and k_3 to vary independently yielded a K_{BD} value of 0.67.

Supplementary Tables

Supplementary Table 1 – Comparison of intramolecular distances in the closed state

*Apparent distances between the dye reporters were calculated for the closed state based on FRET efficiencies (see Methods).

**FRET-weighted distances were calculated using the program FPS of Seidel and coworkers[4], which takes into account the distribution of conformers of the dyes when attached to the protein. The distances calculated from experimental FRET efficiencies are in reasonable agreement with the distances estimated from the crystal structure of the electron-transfer intermediate mimic.

Donor position	Calculated Förster distance for donor-acceptor pair on TbQSOX (Å)	Apparent distances between donor and acceptor in the closed state according to smFRET experiments (Å)*	FRET-weighted distance estimated according to crystal structure (PDB ID 3QD9) of the closed TbQSOX conformation (Å)**
77	53	54	57
116	56	36	39
142	54	50	50
147	56	40	42
160	55	50	58
189	54	58	69

Supplementary Table 2 – Rate constants and species ratios obtained from global fitting the smFRET and turnover data to the model

Values in the top row of the cell were obtained from the fit. According to an F test (see Supplementary Methods), the values are within the ranges reported in the bottom row with a confidence level of 95%.

*The term K_A was put into the fit as a fixed value, obtained from the smFRET experiment in the absence of substrate.

**The term $\frac{1}{K_{CD}}$ obtained from the fits was very small, and so was set to zero. Setting $\frac{1}{K_{CD}}$ to zero did not change the values obtained for the other parameters.

#The rate constant for FAD reduction was put into the fit as a fixed value obtained from previous studies[5], in which FAD reduction rates were measured up to ~3 mM DTT. At this DTT concentration, most of the enzyme molecules are closed (Fig. 5b), and so the previously reported rate constant was considered to characterize FAD reduction from a closed conformation (*i.e.,* k_2).

##See Supplementary Fig. 15.

enzyme	fit	K_A*	k_1 (sec^{-1}mM^{-1})	k_2 (sec^{-1})	k_3 (sec^{-1})	K_{BD}	$1/K_{CD}$	K_D
wild-type	Previous report[2]	-	460	17.9 (FAD reduction)	-	-	-	-
	(1)+(2)	14	390<br>290-560	19.0<br>16.8-21.0	19.0<br>17.6-20.5	2.0<br>1.7-2.4	0**	0.008<br>0.005-0.013
Ala71Pro	Previous report[5]	-	-	7.1 (FAD reduction)	-	-	-	-
	(1)+(2)	3	75<br>60-95	7.1#	15.8<br>14.3-17.6	2.4<br>2.1-2.7	0.11<br>0.02-0.22	0.004<br>0.003-0.006
Arg382Ala	(1)+(2)	28	200<br>140-270	9.4<br>7.9-11.0	10.2<br>9.6-10.9	1.15<br>1.0-1.4	0**	0.019<br>0.011-0.031
Arg74Ala	(1)+(2)	65	250<br>160-400	12.0<br>9.6-15.7	N/A##	0.52<br>0.43-0.66	0**	0.010<br><0.030
Val379Ala	(1)+(2)	32	300<br>210-430	9.5<br>8.5-10.5	5.0<br>4.4-5.7	1.9<br>1.6-2.3	0**	0.003<br>0.002-0.005

Supplementary Methods

Estimation of errors for kinetic and equilibrium constants obtained from global fits. We performed a rigorous error analysis to ensure that the parameter values are robust and to obtain their confidence intervals. In this analysis, each parameter in turn was constrained multiple times at various values within a range around the value obtained from the unconstrained fit, while all other parameters were allowed to change. The χ^2 statistic was plotted for each value of the constrained parameter. An F-test was used to find when the null hypothesis that the constrained model is similar to the original model could be rejected with a P<5%. An example for such an analysis for the K_{BD} parameter of the wild-type enzyme is presented below:

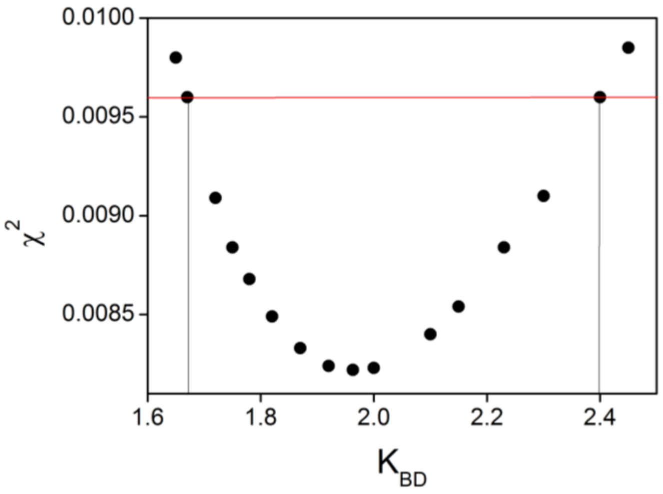

The red line is the value of χ2 above which the null hypothesis is rejected with a P<5%. The two parameter values in the plot that correspond to this upper limit χ^2 value (designated by black vertical lines), define the 95% confidence interval for the parameter and are reported in Supplementary Table 2. This way of calculating the confidence interval automatically takes into account correlations between parameters, and hence does not necessarily lead to a symmetric interval around the minimum. For testing the model in the boundaries of the parameters, see Supplementary Fig. 13.

Supplementary References

1. Gopich, I.V. & Szabo, A. Decoding the pattern of photon colors in single-molecule FRET. *J. Phys. Chem. B.* **113**, 10965–10973 (2009).
2. Kodali, V. K. & Thorpe, C. Quiescin sulfhydryl oxidase from Trypanosoma brucei: catalytic activity and mechanism of a QSOX family member with a single thioredoxin domain. *Biochem.* **49**, 2075-2085 (2010).
3. Alon, A. *et al*. The dynamic disulphide relay of quiescin sulphydryl oxidase. *Nature* **488**, 414-418 (2012).
4. Kalinin, S. *et al*. A toolkit and benchmark study for FRET-restrained high-precision structural modeling. *Nature Meth*. **9**, 1218-1225 (2012).
5. Israel, B. A., Kodali, V. K. & Thorpe, C. Going through the barrier: coupled disulfide exchange reactions promote efficient catalysis in quiescin sulfhydryl oxidase. *J. Biol. Chem.* **289**, 5274-5284 (2014).
